# Evaluation of the MAScIR SARS-CoV-2 M Kit 2.0 on the SARS-CoV-2 Infection

**DOI:** 10.1155/2023/9313666

**Published:** 2023-02-07

**Authors:** Amal Zouaki, Hakima Kabbaj, Ghizlane El Amin, Mouna Ouadghiri, Bouchra Belefquih, Azeddine Ibrahimi, Myriam Seffar

**Affiliations:** ^1^Central Laboratory of Virology (LCV), Ibn Sina University Hospitalo Center (CHUIS), Faculty of Medicine and Pharmacy of Rabat, Mohammed V University, Rabat, Morocco; ^2^Laboratory of Medical Biotechnology (MedBiotech), Bioinova Research Center, Faculty of Medicine and Pharmacy of Rabat, Mohammed V University, Rabat, Morocco; ^3^Laboratory of Medical Analysis Biolife, Temara, Morocco

## Abstract

SARS-CoV-2 is a major public health problem worldwide. Since its emergence, several diagnostic kits have been developed to ensure rapid patient management. The aim of our study is to check the performance of the new Moroccan SARS-CoV-2 detection kit: MAScIR SARS-CoV-2 M 2.0. The following parameters were studied: repeatability, reproducibility, analytical specificity, analytical sensitivity, and comparison with the GeneFinder™ COVID-19 Plus RealAmp Kit. In addition, an external quality evaluation comprising five specimens was carried out as part of an international program for the external quality evaluation of sublaboratories of the WHO and the Laboratory Office of the National Institute of Hygiene of Morocco. The results of all parameters studied showed an analytical performance that complied with the requirements of the method verification/validation protocol adopted by the Central Laboratory of Virology and met the recommendations of COFRAC (French Accreditation Committee). During the current study, the sequencing of some randomly selected positive samples was performed, among which the carriers of the Alpha variant, the Delta variant, and the Omicron variant were detected. These results allowed us to deduce that this kit was valid for detecting these three variants.

## 1. Introduction

Severe acute respiratory syndrome coronavirus 2 (SARS-CoV-2), responsible for coronavirus disease 2019 (COVID-19), first appeared in December 2019 in Wuhan, China [1, 2]. Its rapid transmission and widespread spread have allowed it to rapidly evolve into a pandemic.

It is an enveloped, nonsegmented, positive-sense single-stranded RNA virus belonging to the Coronaviridae family and the order Nidovirals [3]. The coding part of its genome consists essentially of two regions: the first region, which represents two-thirds of the genome, codes for the nonstructural proteins of the replication/transcription complex (ORF1a and ORF1b), and the second region, which represents the third of the genome, codes for the structural proteins of the virus (spike, envelope, membrane, and nucleocapsid) [3–5].

The diagnosis of SARS-CoV-2 is essentially based on molecular methods and qualitative RT-PCR, which allow the simultaneous detection of at least one SARS-CoV-2 target and internal control (https://www.sfm-microbiologie.org/wp-content/uploads/2021/02/LISTE-RAPPORTS-TESTS-MOLECULAIRES_130121.pdf). Many kits are available, and the most commonly used viral targets are located in the genes S (spike), E (envelope), N (nucleocapsid), and RdRp (ORF1a-dependent RNA polymerase) [4, 6]. The main objective of this study was to evaluate the performance of the new Moroccan MAScIR SARS-CoV-2 M 2.0 kit.

## 2. Materials and Methods

### 2.1. Specimens

This study uses the method verification/validation procedure of the Central Laboratory of Virology (LCV) of the University Hospital Ibn Sina, Rabat, which meets the recommendations of the French Committee for Accreditation (COFRAC), and uses qualitative tests similar to quantitative tests on the basis of Ct. Specimens were taken from patients by nasopharyngeal or oropharyngeal swabs, which were then sent to the LCV in a viral transport medium for qualitative detection of the SARS-CoV-2 genome. The retrospective portion of the study was performed on specimens held at −80°C. The stability of the specimens was verified by the results of their cellular internal control (IC) that met the supplier's recommendations with a value of cycle threshold (Ct) less than 35.

### 2.2. Nucleic Acid Extraction

Automated nucleic acid extraction was performed on prefilled plates (from 16 wells) with the BIOER extractor. In an extraction plate, 10 *μ*L of proteinase *K* was deposited and 300 *μ*L of the patient sample was deposited. The number of patients treated in each extraction series was 32 (simultaneous extraction of two columns) with a total time of 35 min.

### 2.3. Nucleic Acid Amplification

#### 2.3.1. New MAScIR SARS-CoV-2 M 2.0 Kit Analytical Procedure

It is a qualitative triplex in vitro amplification test, based on one-step reverse transcription polymerase chain reaction (RT-qPCR) and whose targets were two viral genes of SARS-CoV-2 (RdRp and S) and an internal control of human cell origin. All three targets were treated in the same reaction well. Each reaction mixture contains 2.5 *μ*L of the enzyme mix (enzyme cocktail, dNTP, and reaction buffer), 1 *μ*L of the primer and probe mix, and 6.5 *μ*L of viral extract from patient samples (eluate). Positive control, negative control, and negative extraction control were used in each series of samples.

The amplification was performed on three thermal cyclers, ABI 7500 Applied Biosystems, QuantStudio™ 5 (QS5) Applied Biosystems, and Exicycler™ 96 Bioneer. The amplification program was as follows: a reverse transcription step at 50°C for 5 minutes, then an activation step at 95°C for 20 seconds, followed by a succession of 40 cycles of denaturation-hybridization-elongation (denaturation occurs at 95°C for 3 seconds and hybridization and elongation at 60°C for 30 seconds). The fluorophores of the probes used in this kit were FAM for RdRp, Cy5 for S, and VIC for CI [7]. The total duration of the PCR on the QS5 was 56 minutes.

#### 2.3.2. GeneFinder™ COVID-19 Plus RealAmp Reference Kit Analytical Procedure

The GeneFinder™ COVID-19 Plus RealAmp Kit was an *in vitro* amplification test based on real-time polymerase chain reaction (RT-qPCR). It allows the detection of three viral targets (RdRp, N, and E) and internal cellular control. In each reaction well, 5 *μ*L of the patient sample eluate was mixed with 15 *μ*L of the master mix. Positive and negative controls were also used in each series of samples.

The amplification, performed on the three aforementioned thermal cyclers, includes the following steps: a reverse transcription step at 50°C for 20 minutes and then an activation step at 95°C for 5 minutes, followed by a succession of 45 denaturation-hybridization-elongation cycles (denaturing occurs at 95°C for 15 seconds and hybridization and elongation occur at 60°C for 60 seconds) [8]. The total duration of the PCR on the QS5 thermal cycler is 120 minutes.

Over an eight-month period, 171,548 tests were conducted with the GeneFinder™ COVID-19 Plus Real Amp Kit in our laboratory.

### 2.4. Validation Step of the Results of the MAScIR SARS-CoV-2 M 2.0 Kit

The validation step was divided into three parts. The first step involved the validation of the negative control, the negative extraction control, and the positive control. For negative checks, all targets have been negative, while for positive controls, all targets have been positive with a Ct value of 22 ± 2 for the RdRp, 19 ± 2 for S, and 24 ± 2 for the CI, according to the data in the manual of the MAScIR SARS-CoV-2 M 2.0.

The second step was the validation of each patient's internal control. This control, which was of cellular origin, allows controlling the quality of the sample since its collection, as well as the proper functioning of the extraction and amplification steps and the absence of inhibition. The CI of the samples has been positive, with a Ct< 35.

After the validation of the controls, the patients' results were read. According to the recommendations of the kit leaflet and the recommendations of the SFM [9], a sample was considered positive if one or both targets (RdRp and S) had a Ct less than 30, low positive if the Ct value was between 31 and 36, and negative if the Ct value is >37. All control and patient results were collected in a reading sheet and validated on the Laboratory Information System (SIL) (e-Labs, ENOVA Research and Technology). The outcome report provided to the patient and/or attending physician mentions the presence or absence of SARS-CoV-2 RNA.

### 2.5. Protocol of the Study

The repeatability study was carried out on three pools of samples of different concentrations on the basis of Ct (one pool of high concentration, one pool of medium concentration, and one pool of low concentration). It was performed on the QuantStudio™ 5 Applied Biosystems thermal cycler. Each sample had five runs on the same day, in the same series, with the same working conditions and operators, the same procedure, and the same batch of reagents. The analytical objective of the repeatability test recommended by the supplier was a CV < 5%.

The intermediate precision test was studied on positive controls. The results of the positive checks of the same batch, carried out in each series and over several days on two different thermal cyclers by different operators, were collected on a sheet and then processed on the EVM. The limit CV value was 6.66% (calculated according to the formula CV repeatability = CV reproducibility × 0.75).

Moreover, to evaluate the analytical specificity of the test, 19 positive samples for a respiratory virus different from SARS-CoV-2, whose diagnosis was made by PCR in real-time using GeneXpert (retrospective samples from 2019) or FilmArray BIOFIRE (prospective samples from 2020–21) have been analyzed by the new MAScIR SARS-CoV-2 M 2.0 kit.

In addition, the analytical sensitivity of the MAScIR SARS-CoV-2 M 2.0 kit was investigated through a series of five dilutions of the positive control of the kit (Ct RdRp: 16, Ct S: 18, and Ct CI: 17) with a diluent containing transfer RNA at a concentration of 10 ng/*μ*L.

Furthermore, an external quality assessment (EQA) of this MAScIR SARS-CoV-2 M 2.0 kit, comprising five samples, was carried out in January 2021 as part of an international program of external quality assessment of sublaboratories of National Institute of Hygiene (INH) of Morocco. Furthermore, to monitor the genetic evolution of the virus, several sequencing units of SARS-CoV-2 strains have been set up in Morocco. In this context, several positive samples, diagnosed by the MAScIR SARS-CoV-2 M 2.0 at the LCV, were randomly selected and sequenced at the Medical Biotechnology Laboratory of the Faculty of Medicine and Pharmacy of Rabat using Ion S5 (Ion S5 next-generation sequencing technology).

Finally, the comparison between the GeneFinder™ COVID-19 Plus RealAmp Kit and the MAScIR SARS-CoV-2 M 2.0 kit was performed on 61 samples on ABI 7500 Applied Biosystems, 56 samples on QuantStudio™ 5 Dx Applied Biosystems, and 45 samples on Exicycler™ 96 Bioneer.

All results of this method validation were processed using the Middleware EVM Byg Informatique (EVM). Data processing was based on the calculation of the Ct mean, standard deviation, and coefficient of variation for each parameter. The results were later converted into a graph of the distribution of values around the mean and into a Levey–Jenning curve. The precision of each calculation was expressed as a function of statistical measures of imprecision (standard deviation and coefficient of variation). Data from the comparison between the two kits were processed using the Bland–Altman concordance method on EVM.

## 3. Results

### 3.1. Analytical Performance

#### 3.1.1. Repeatability

The average Ct of the RdRp target is 33.24 for the low-concentration pool, 27.61 for the medium-concentration pool, and 14.52 for the high-concentration pool. The CVs of the three concentration levels are, respectively, 2.20%, 2.29%, and 2.69%. For target S, the Ct average is 34.63 for the low-concentration pool, 28.69 for the medium-concentration pool, and 16.02 for the high-concentration pool, while the CV values are 3.06%, 1.76%, and 5.44%, respectively. Only the CV value of the S target at the high concentration level is limited to the value recommended by the supplier (Table 1).

#### 3.1.2. Intermediate Precision

**Figure 1:**
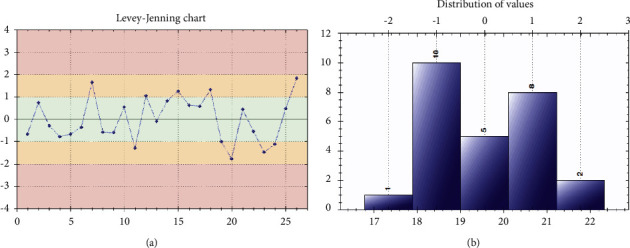
Levey–Jenning chart (a) and distribution of values (b) of intermediate precision test results of MAScIR SARS-CoV-2 kit M 2.0. (target RdRp, QS5 thermal cycler).

On the QS5 thermocycler, the Ct mean value of the targets (out of a total of 26 values) is 19.56 for the RdRp and 20.64 for the S gene, while on the EXICYCLER thermocycler, the Ct mean of the targets (out of 13 values) is 21.62 for the RdRp and 21.04 for the S gene. All CV values in our series are higher than those of our supplier but remain below the recommended target CV value. For the overall reproducibility of the 2 thermocyclers, the average Ct of the targets is 20.25 for RdRp and 20.78 for S. The RdRp CV is limited to the target recommended by the supplier (Table 1, Figure 1).

#### 3.1.3. Analytical Specificity and Sensitivity

The number of samples distributed by pathogen was Influenza A (1), Influenza B (2), Respiratory syncytial virus (RSV) (5), Parainfluenza virus 3 (2), Parainfluenza virus 4 (2), Coronavirus 229E (1), Coronavirus OC43 (2), Rhinovirus/Enterovirus (6), and Adenovirus (1). All of these samples were nonreactive for SARS-CoV-2 (Table 2).

**Figure 2:**
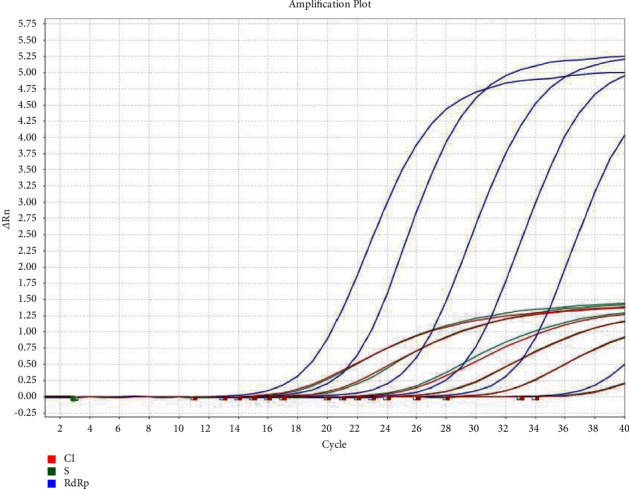
Analytical sensitivity test result of the MAScIR SARS-CoV-2 M 2.0 kit. Target S, green; target RdRp, blue; IC, red.

For analytical sensitivity, the Ct results of the five dilutions of the positive control were as follows: for RdRp, the values were 18, 22, 26, 30, and 37, respectively, while for S, the results were 20, 24, 28, 32, and 38, respectively. The resulting lower detection limit, defined as the highest detectable Ct value, was a Ct of 37 for RdRp and 38 for S (Figure 2).

#### 3.1.4. Interlaboratory Accuracy/Comparison

The five EQA results were compliant. However, the Ct comparison and measurement accuracy calculation were not possible as the results provided to us did not specify the Ct values of the targets (Table 3).

#### 3.1.5. Comparison between SARS-CoV-2 M 2.0 MAScIR Kit and COVID-19 Plus RealAmp GeneFinder™ Reference Kit

**Figure 3:**
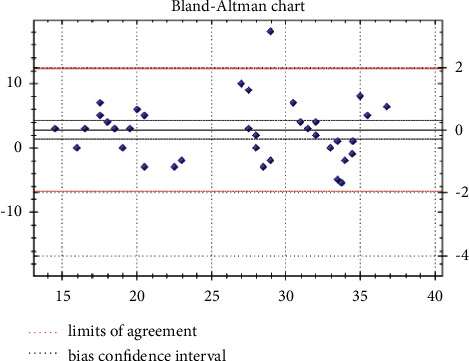
The analysis of RdRp results using the Bland and Altman method (results on QS5 thermal cycler).

The qualitative results of this comparison were broadly consistent. No discrepancies with clinical impact were detected (Table 4). For the quantitative results, based solely on the Ct value of the RdRp (the only common target between the two kits), the application of the Bland–Altman concordance method objectified a single value greater than the upper concordance limit (and this on the three thermal cyclers used), which was the sample No. 11 (Figure 3, Table 5). The sensitivity and specificity of the MAScIR SARS-CoV-2 M 2.0 kit were estimated at 100%, using the GeneFinder™ COVID-19 Plus test as a reference.

### 3.2. Detection of SARS-CoV-2 Variants Present in Morocco

The sequencing of positive samples allowed the detection of cases carrying one of the variants of SARS-CoV-2 with identical Ct for S and RdRp (the Alpha variant, the Delta variant, or the Omicron variant). The results of some cases are given in Table 6, and all data are available on GISAID.

## 4. Discussion

After SARS-CoV-1 (which was responsible for an outbreak in China in 2002-2003) and MERS-CoV (which spread to 27 countries between 2012 and 2018), the new SARS-CoV-2 was responsible for the global pandemic that started in December 2019 in Wuhan, China, Hubei Province, following the appearance of several contacts with similar symptoms [3, 10]. Until May 19, 2022, this pandemic was responsible for more than 520 million cases worldwide, including 6.27 million deaths. In Morocco, the number of cumulative cases over the same period was up to 1.16 million, including 16.075 million cases of death [11].

To address this emergency, many measures have been put in place to limit the spread of SARS-CoV-2, including diagnostic tools to identify people with SARS-CoV-2, ensure their clinical and therapeutic follow-up, and prevent the transmission of the virus.

In this context, the Moroccan Foundation for Advanced Science Innovation and Research (MAScIR) was able to develop a new Moroccan kit for the qualitative detection of RNA of the SARS-CoV-2, based on RT-qPCR technology [7, 12]. The first version of this kit, MAScIR SARS-CoV-2 kit 1.0, was evaluated by the Institute Pasteur in Paris, French National Reference Centre for Respiratory Infections. It was a test that allowed the detection of three viral targets of SARS-CoV-2 (RdRp, S, and E) and cell CI, which required the treatment of each sample in two different wells with the use of two reaction mixes [12]. This version has been optimized by the introduction of the second version, MAScIR SARS-CoV-2 kit M 2.0, CE-IVD, the subject of the present work, which detects two viral targets (RdRp and S) and the CI with the advantage of treating each sample in a single reaction well.

The accuracy study of this kit was consistent with that of the supplier. The repeatability test as well as the intermediate fidelity test have coefficients of variation higher than the supplier's results but remain below the recommended CV value [7].

Regarding the evaluation of analytical specificity, the results were excellent, with 100% specificity. All samples included, positive for one or more respiratory viruses, including viruses belonging to the Coronaviridae family, were negative for SARS-CoV-2 with the MAScIR SARS-CoV-2 kit M 2.0. No cross-reaction was detected. According to our literature review, the RdRp and S genes (which are the targets in our kit), along with the N genes of SARS-CoV-2, are known for their higher specificity [13].

Comparison of the results with the reference kit (GeneFinder™ COVID-19 Plus RealAmp Kit) was compliant. All negative samples using the reference kit were negative using the MAScIR SARS-CoV-2 M 2.0 kit, and all positive samples were positive. However, some highly positive samples using the reference kit had a low positive result using the MAScIR SARS-CoV-2 M 2.0 kit (2/38 on ABI 7500, 2/34 on QS5 Dx, and 3/24 on Exicycler 96). Conversely, some low positive samples had a strong positive result using MAScIR SARS-CoV-2 M 2.0 kit (8/14 on ABI 7500, 7/13 on QS5 Dx, and 7/12 on Exicycler 96) (Table 5). These differences could be explained by a partial degradation of viral RNA during conservation and/or variable extraction performance. Furthermore, the MAScIR SARS-CoV-2 M 2.0 kit's amplification duration is shorter (56 minutes versus 120 minutes) compared to the reference kit. This represents a significant time advantage, allowing a faster result and a quicker management of exam requests, especially during waves of cases [7, 8].

In addition, the analysis of RdRp results using the Bland and Altman method shows a good correlation between the two methods except for a sample that gives an out-of-bounds result (Ct from RdRp: 38 using the reference kit vs Ct from RdRp being between 20 and 21 using the evaluated kit) (Table 5). In general, these results have no clinical impact, as the test under study is qualitative. Moreover, the external evaluation by the EEQ confirmed the reliability of the results.

On the other hand, for the GeneFinder™ COVID-19 Plus Real Amp Kit, the most sensitive target was the N gene. Out of the 52 positive samples included in the comparison test, RdRp and E targets were not detected in 2/52 and 4/52 samples, respectively, unlike the N target that was detected in all of these samples. This could be explained by the higher expression of ARNm transcripts of the N gene compared to the other genes, which provides a higher starting amount of template. This was discussed by Karen Yanson et al. in their article [14]. However, other articles have demonstrated that kits targeting the E gene are considered the most sensitive [13]. For the MAScIR SARS-CoV-2 M 2.0 kit, the most sensitive target is the S gene that was detected in all samples regardless of the thermal cycler used. However, the detection of the RdRp gene varies depending on the thermal cycler: with the QS5, the target was detected in all samples, while with the ABI and Exicycler, the target was not detected in 1/52 and 7/52 samples, respectively. Moreover, the S target of the MAScIR SARS-CoV-2 M 2.0 kit is not affected by the mutation of the Alpha, Delta, or Omicron variant since the Ct of the RdRp and S genes were identical, which was confirmed by the sequencing of our samples (Tables 5 and 6) [15, 16]. Our entire dataset has been published on the Mendeley Data Repository [17].

To date, a single retrospective study, recently published and carried out in our laboratory, evaluated the FilmArray BioFire RP2.1 (Respiratory 2.1 Panel) kit using our MAScIR SARS-CoV-2 M 2.0 kit as a reference kit [18]. In this study, there were no discordant results between MAScIR and FilmArray BioFire RP2.1 with regards to negative samples. However, 21 of the 80 positive SARS-CoV-2 samples on FilmArray had discordant results on MAScIR SARS-CoV-2 M 2.0. Eleven of these were tested with another kit, revealing positive results in six cases [18]. This has been explained by the difference in the limit of detection (LoD) between the two kits: 160 copies/mL for FilmArray and 500 copies/mL for MAScIR [7, 18, 19]. Moreover, the sensitivity of the first MAScIR SARS-CoV-2 kit 1.0 has been validated by the National Respiratory Infections Reference Centre, as mentioned above. It is to be noted that three studies provided data for the GeneFinder™ COVID-19 Plus RealAmp [20–22].

## 5. Conclusion

Since the beginning of this pandemic, many SARS-CoV-2 detection PCR kits have been developed. In this context, the MOLDIAG company, created by the MAScIR Foundation, developed the first qualitative PCR kit designed and manufactured in Morocco for the detection of SARS-CoV-2. This study, carried out by the LCV team, has made it possible to verify the performance of this new MAScIR SARS-CoV-2 M 2.0 kit. The results of the parameters studied are consistent with the supplier's recommendations and are consistent with those of the reference method. This kit also has certain advantages: the price, the manufacture, and the availability in Morocco, as well as the amplification time (56 min), which is reduced compared to the GeneFinder™ COVID-19 Plus RealAmp Kit, which allowed to adopt it as a test for detection of the routine SARS-CoV-2 in high-throughput labs.

## Figures and Tables

**Table 1:** Repeatability and intermediate precision tests results of MAScIR SARS-CoV-2 kit M 2.0.

	Target	Number of values (*N*)	Mean of Ct	SD	CV%	CV%	Conclusion
						Supplier notice	
Repeatability							
Concentration of samples							
High concentration	RdRp	5	14.52	0.39	2.69	1.56	Compliant
	S		16.02	0.87	5.44	1.22	Limit
	CI		21.94	0.33	1.49	1.69	Compliant
Medium concentration	RdRp	5	27.61	0.63	2.29	1.70	Compliant
	S		28.69	0.50	1.76	1.86	Compliant
	CI		25.15	0.45	1.77	2.48	Compliant
Low concentration	RdRp	5	33.24	0.73	2.20	1.70	Compliant
	S		34.63	1.06	3.06	1.86	Compliant
	CI		24.19	0.90	3.73	2.48	Compliant

Intermediate precision							
Thermal cycler							
QS5	RdRp	26	19.56	1.11	5.67	2.54	Compliant
	S		20.64	1.09	5.27	1.87	Compliant
	CI		21.95	1.18	5.39	3.49	Compliant
Exicycler	RdRp	13	21.62	0.75	3.46	2.54	Compliant
	S		21.04	0.87	4.11	1.87	Compliant
	CI		21.05	0.98	4.67	3.49	Compliant
Global intermediate precision (QS5 + Exicycler)	RdRp	39	20.25	1.40	6.90	2.54	Limit
	S		20.78	1.03	4.93	1.87	Compliant
	CI		21.65	1.19	5.49	3.49	Compliant

CV, coefficient of variation; SD, standard deviation.

**Table 2:** Analytical specificity test result of the MAScIR SARS-CoV-2 M 2.0 kit.

Date	PCR GeneXpert results	PCR SARS-CoV-2 MAScIR kit 2.0 triplex results (QS5)
22/12/2020	Influenza B	Internal control: positive SARS-CoV-2: negative
	Influenza A	
	Influenza B	
	Respiratory syncitial virus	
	Respiratory syncitial virus	
	Respiratory syncitial virus	
	Respiratory syncitial virus	
	Respiratory syncitial virus	

Date	FilmArray BIOFIRE results	PCR SARS-CoV-2 MAScIR kit 2.0 triplex results (QS5)

01/01/2021	Parainfluenza virus 3	Internal control: positive SARS-CoV-2: negative
12/01/2021	Coronavirus 229E	
26/01/2021	Coronavirus OC43	
26/01/2021	Rhinovirus/enterovirus	
30/01/2021	Rhinovirus/enterovirus	
01/02/2021	Rhinovirus/enterovirus	
15/02/2021	Adenovirus Parainfluenza virus 4	
21/02/2021	Coronavirus OC43	
05/03/2021	Rhinovirus/enterovirus Parainfluenza virus 4	
06/03/2021	Rhinovirus/enterovirus	
20/03/2021	Rhinovirus/enterovirus Parainfluenza virus 3	

**Table 3:** Results of the five EQA (expected results and our laboratory results) (BIOER extractor, QS5 thermal cycler).

	WHO-SC 20-01		WHO-SC 20-02		WHO-SC 20-03		WHO-SC 20-04		WHO-SC 20-05	
	ER	R	ER	R	ER	R	ER	R	ER	R
S target	D	27.00	D	21.00	ND	ND	ND	ND	D	31.00
RdRp target	D	26.00	D	20.00	ND	ND	ND	ND	D	30.00
Interpretation	P	P	P	P	N	N	N	N	P	P

ER, expected result; R, our laboratory result; N, negative; P, positive; D, detected; ND, not detected.

**Table 4:** Analysis of qualitative results.

Thermal cycler	GeneFinder^™^ COVID-19 Plus RealAmp (reference)		MAScIR SARS-CoV-2 kit 2.0	
	Result	Number of samples	Result	Number of samples
ABI 7500 applied biosystems	P	38	P	36
			LP	2
	LP	14	P	8
			LP	6
	N	9	N	9

QuantStudio™ 5 applied biosystem	P	34	P	32
			LP	2
	LP	13	P	7
			LP	6
	N	9	N	9

Exicycler™ 96 Bioneer	P	24	P	21
			LP	3
	LP	12	P	7
			LP	5
	N	9	N	9

P, positive (target Ct < 30); LP, low positive (target Ct from 31 to 36); N, negative (target Ct > 37).

**Table 5:** Comparison of PCR SARS-CoV-2 results between SARS-CoV-2 M 2.0 MAScIR kit and COVID-19 Plus RealAmp GeneFinder™ reference kit.

Samples	SARS-CoV-2 M 2.0 MAScIR Kit						COVID-19 Plus RealAmp GeneFinder^TM^Kit
	Extracteur	Thermal cycler	Target RdRp	Target S	IC	Interpretation	Result
1	BIOER	QS5	24	24	26	P	P
		ABI	25	24	30		
		Exicycler	24	24	26		

2	BIOER	QS5	22	23	24	P	P
		ABI	23	22	27		
		Exicycler	23	23	24		

3	BIOER	QS5	35	34	25	LP	LP
		ABI	25	33	28	P	
		Exicycler	31	30	24		

4	BIOER	QS5	36.5	32	27	LP	P
		ABI		32	31		
		Exicycler	±35	32	27		

5	BIOER	QS5	36	34	25	LP	P
		ABI	35	33	27		
		Exicycler		f34	24		

6	BIOER	QS5	33	32	25	LP	LP
		ABI	33	32	27		
		Exicycler	32	32	25		

7	BIOER	QS5	33	33	26	LP	LP
		ABI	32	31	27		
		Exicycler		f32	25		

8	BIOER	QS5	30	30	26	P	LP
		ABI	31	30	28		
		Exicycler	f31	30	25		

9	BIOER	QS5	23	24	25	P	LP
		ABI	24	23	28		
		Exicycler	23	23	24		

10	BIOER	QS5	15	16	21	P	P
		ABI	16	15	24		
		Exicycler	15	15	20		

11	BIOER	QS5	20	20	24	P	LP
		ABI	21	20	26		
		Exicycler	20	20	22		

12	BIOER	QS5	23	24	22	P	P
		ABI	25	23	23		
		Exicycler	24	23	21		

13	BIOER	QS5	16	16	23	P	P
		ABI	17	16	26		
		Exicycler	16	16	22		

14	BIOER	QS5	22	22	23	P	P
		ABI	23	21	25		
		Exicycler	22	22	22		

15	BIOER	QS5	—	—	27	N	N
		ABI	—	—	30		
		Exicycler	—	—	26		

16	BIOER	QS5	—	—	24	N	N
		ABI	—	—	26		
		Exicycler	—	—	23		

17	BIOER	QS5	—	—	26	N	N
		ABI	—	—	28		
		Exicycler	—	—	25		

18	BIOER	QS5	—	—	25	N	N
		ABI	—	—	27		
		Exicycler	—	—	24		

19	BIOER	QS5	—	—	25	N	N
		ABI	—	—	27		
		Exicycler	—	—	23		

20	BIOER	QS5	—	—	27	N	N
		ABI	—	—	28		
		Exicycler	—	—	25		

21	BIOER	QS5	—	—	26	N	N
		ABI	—	—	28		
		Exicycler	—	—	25		

22	BIOER	QS5	—	—	24	N	N
		ABI	—	—	26		
		Exicycler	—	—	23		

23	BIOER	QS5	16	16	24	P	P
		ABI	16	15	27		
		Exicycler	15	15	22		

24	BIOER	QS5	28	28	25	P	P
		ABI	29	27	27		
		Exicycler	28	28	25		

25	BIOER	QS5	30	29	24	P	LP
		ABI	30	29	25		
		Exicycler	30	29	23		

26	BIOER	QS5	26	27	23	P	P
		ABI	28	27	23		
		Exicycler	28	27	21		

27	BIOER	QS5	13	13	24	P	P
		ABI	13	13	26		
		Exicycler	13	12	21		

28	BIOER	QS5	18	19	26	P	P
		ABI	19	18	28		
		Exicycler	18	17	23		

29	BIOER	QS5	19	19	23	P	P
		ABI	20	18	25		
		Exicycler	19	19	21		

30	BIOER	QS5	30	30	24	P	P
		ABI	31	30	26		
		Exicycler	31	30	23		

31	BIOER	QS5	17	18	26	P	P
		ABI	18	16	27		
		Exicycler	17	17	23		

32	BIOER	QS5	24	19	24	P	P
		ABI	23	18	25		
		Exicycler	19	19	23		

33	BIOER	QS5	17	17	24	P	P
		ABI	18	16	25		
		Exicycler	23	17	23		

34	BIOER	QS5	23	24	25	P	P
		ABI	24	23	26		
		Exicycler	23	24	24		

35	BIOER	QS5	33	33	23	LP	LP
		ABI	35	39	25		
		Exicycler		f33	22		

36	BIOER	QS5	30	26	24	P	P
		ABI	31	25	26		
		Exicycler	26	26	24		

37	BIOER	QS5	31	32	23	P	P
		ABI	32	30	25		
		Exicycler	—	31	23	LP	

38	BIOER	QS5	14	15	23	P	P
		ABI	15	14	27		
		Exicycler	14	14	22		

39	BIOER	QS5	18	18	22	P	P
		ABI	18	16	22		
		Exicycler	18	18	21		

40	BIOER	QS5	31	31	22	P	LP
		ABI	31	30	22		
		Exicycler	±32	30	21		

41	BIOER	QS5	34	33	28	LP	LP
		ABI	36	33	30		
		Exicycler	—	f34	27		

42	BIOER	QS5	34.9	33	25	LP	LP
		ABI	34	33	27		
		Exicycler	f33	f33	24		

43	BIOER	QS5	33.6	32	24	P	P
		ABI	33	31	25		
		Exicycler	32	31	23		

44	BIOER	QS5	27	23	24	P	LP
		ABI	28	23	26		
		Exicycler	23	23	23		

45	BIOER	QS5	—	36.9	26	N	N
		ABI	—	37	28		
		Exicycler	—	—	25		

46	BIOER	QS5	15	16	24	P	P
		ABI	16	15	27		

47	BIOER	QS5	28	28	25	P	P
		ABI	28	27	27		

48	BIOER	QS5	29	29	24	P	LP
		ABI	30	29	25		

49	BIOER	QS5	27	27	23	P	P
		ABI	27	26	23		

50	BIOER	QS5	13	13	24	P	P
		ABI	13	12	25		

51	BIOER	QS5	18	19	26	P	P
		ABI	19	18	28		

52	BIOER	QS5	19	19	24	P	P
		ABI	20	19	25		

53	BIOER	QS5	30	30	24	P	P
		ABI	31	29	26		

54	BIOER	QS5	18	18	26	P	P
		ABI	18	17	28		

55	BIOER	QS5	24	19	24	P	P
		ABI	23	18	25		

56	BIOER	QS5	17	17	23	P	P
		ABI	17	16	24		

57	BIOER	ABI	24	23	26	P	P

58	BIOER	ABI	34	32	23	LP	LP

59	BIOER	ABI	31	25	26	P	P

60	BIOER	ABI	33	30	24	P	P

61	BIOER	ABI	15	14	25	P	P

P, positive (target Ct < 30); LP, low positive (target Ct from 31 to 36); N, negative (target Ct > 37).

**Table 6:** References on GISAID of some cases belonging to the line Alpha, Delta, or Omicron diagnosed at the LCV and sequenced at the Laboratory of Biotechnology of FMP, Rabat.

Virus name	Accession ID	Age	Sex	Thermal cycler	PCR MAScIR (Central Laboratory of Virology-CHUIS)		Variant (ion torrent, Laboratory of Medical Biotechnology, FMP, Rabat)
					Ct RdRp target	Ct S target	
hCoV-19/Morocco/FMP-288/2021	EPI_ISL_1905079	69	M	QS5	15	15	VOC 202012/01 GRY (B.1.1.7) first detected in the UK
hCoV-19/Morocco/FMP-280/2021	EPI_ISL_1905060	68	F	QS5	23	23	VOC 202012/01 GRY (B.1.1.7) first detected in the UK
hCoV-19/Morocco/FMP-253/2021	EPI_ISL_1904888	26	F	QS5	30	31	VOC 202012/01 GRY (B.1.1.7) first detected in the UK
hCoV-19/Morocco/FMP-256/2021	EPI_ISL_1904887	23	M	QS5	30	30	VOC 202012/01 GRY (B.1.1.7) first detected in the UK
hCoV-19/Morocco/FMP-255/2021	EPI_ISL_1904886	43	F	QS5	31	31	VOC 202012/01 GRY (B.1.1.7) first detected in the UK
hCoV-19/Morocco/FMP-377/2021	EPI_ISL_13961937	41	F	QS5	24	25	AY.112 (Pango v.4.1.2 PLEARN-v1.12), Delta (B.1.617.2-like) (Scorpio)
hCoV-19/Morocco/FMP381/2021	EPI_ISL_13961936	44	M	QS5	26	27	AY.73 (Pango v.4.1.2 PLEARN-v1.12), Delta (B.1.617.2-like) (Scorpio)
hCoV-19/Morocco/FMP362/2021	EPI_ISL_13961935	30	F	QS5	24	23	AY.33 (Pango v.4.1.2 PLEARN-v1.12), delta (B.1.617.2-like) (Scorpio)
hCoV-19/Morocco/FMP-431/2021	EPI_ISL_13961934	59	M	QS5	26	27	BA.1 (Pango v.4.1.2 PLEARN-v1.12), Omicron (BA.1-like) (Scorpio)
hCoV-19/Morocco/FMP424/2021	EPI_ISL_13961933	25	F	QS5	17	18	BA.1 (Pango v.4.1.2 PLEARN-v1.12), Omicron (BA.1-like) (Scorpio)
hCoV-19/Morocco/FMP447/2021	EPI_ISL_13961859	48	F	QS5	25	26	BA.1 (Pango v.4.1.2 PLEARN-v1.12), Omicron (BA.1-like) (Scorpio)
hCoV-19/Morocco/FMP446/2021	EPI_ISL_13961177	62	F	QS5	25	26	BA.1.10 (Pango v.4.1.2 PLEARN-v1.12), Omicron (BA.1-like) (Scorpio)
hCoV-19/Morocco/FMP408/2021	EPI_ISL_13947433	51	M	QS5	29	29	BA.1 (Pango v.4.1.2 PLEARN-v1.12), Omicron (BA.1-like) (Scorpio)

## Data Availability

The data used to support the findings of this study are included within the article.

## References

[1] Shereen M. A., Khan S., Kazmi A., Bashir N., Siddique R. (2020). COVID-19 infection: emergence, transmission, and characteristics of human coronaviruses. *Journal of Advanced Research*.

[2] Holmes E. C., Goldstein S. A., Rasmussen A. L. (2021). The origins of SARS-CoV-2: a critical review. *Cell*.

[3] Vabret A., Ar Gouilh M. (2019). Cytomégalovirus humain in Traité de virologie médicale, 2 ème édition. https://www.sfm-microbiologie.org/wp-content/uploads/2020/04/CHAPITRE38_CORONAVIRUS_TVM2019.pdf.

[4] Hu B., Guo H., Zhou P., Shi Z.-L. (2021). Characteristics of SARS-cov-2 and COVID-19. *Nature Reviews Microbiology*.

[5] Lefeuvre C., Przyrowski É., Apaire-Marchais V. (2020). Aspects virologiques et diagnostic du coronavirus Sars-CoV-2. *Actualités Pharmaceutiques*.

[6] Yüce M., Filiztekin E., Özkaya K. G. (2021). COVID-19 diagnosis -a review of current methods. *Biosensors and Bioelectronics*.

[7] Moldiag MAScIR SARS-CoV-2 M (2021). Kit2.0 pour diagnostic in vitro (IVD). fiche technique, moldiag, Rabat Design Center. https://www.moldiag.ma.

[8] fda.gov (2020). GeneFinder TM COVID-19 Plus RealAmp Kit. Instructions for use, GeneFinder. https://www.fda.gov/media/137116/download.

[9] autorité de santé H. (2020). Avis SFM du 25/09/2020 relatif à l’interprétation de la valeur de Ct (estimation de la charge virale) obtenue en cas de RT-PCR SARS-CoV-2 positive. https://www.sfm-microbiologie.org/wp-content/uploads/2020/09/Avis-SFM-valeur-Ct-excr%C3%A9tion-virale-_-Version-Finale-25092020.pdf.

[10] Wu F., Zhao F., Yu B. (2020). A new coronavirus associated with human respiratory disease in China. *Nature*.

[11] World Health Organization (2021). WHO coronavirus (COVID-19) dashboard. https://covid19.who.int/table.

[12] Sfm-microbiologie (2021). MAScIR_MAScIR-SARS-CoV-2-kit-1.0. https://www.sfm-microbiologie.org/wp-content/uploads/2020/05/MAScIR_MAScIR-SARS-CoV-2-kit-1.0.pdf.

[13] Habibzadeh P., Mofatteh M., Silawi M., Ghavami S., Faghihi M. A. (2021). Molecular diagnostic assays for COVID-19: an overview. *Critical Reviews in Clinical Laboratory Sciences*.

[14] Karen Y., William L., Lori N. (2021). Performance evaluation of the BD SARS-CoV-2 reagents for the BD MAX system. *Journal of Clinical Microbiology*.

[15] Ouadghiri M., Aanniz T., Essabbar A. (2021). Report of SARS-CoV-2 B1.1.7 lineage in Morocco. *Microbiology Resource Announcements*.

[16] El Mazouri S., Bendani H., Boumajdi N. (2022). Report of SARS-CoV-2 BA.1 lineage in Morocco. *Microbiology Resource Announcements*.

[17] Zouaki A., Kabbaj H., El Amin G. (2022). Performance evaluation of the SARS-CoV-2 detection kit: MAScIR SARS-CoV-2 M kit 2.0. https://data.mendeley.com/datasets/3pd8ds983n.

[18] Tazi S., Kabbaj H., Zirar J. (2022). Comparative performance evaluation of film array BioFire RP2.1 and MAScIR 2.0 assays for SARS-CoV-2 detection. *Advances in Virology*.

[19] BioFire Diagnostics and LLC (2020). Biofire Respiratory Panel 2.1 (RP2.1) Instructions for Use. https://docs.biofiredx.com/wp-content/uploads/BFR0000-8303-BioFire-RP2.1-PanelInstructions-for-Use-EUA-EN.pdf.

[20] Farfour E., Lesprit P., Visseaux B. (2020). The allplex 2019-nCoV (Seegene) assay: which performances are for SARS-CoV-2 infection diagnosis?. *European Journal of Clinical Microbiology & Infectious Diseases*.

[21] Ong D. S. Y., Claas E. C. J., Breijer S., Vaessen N. (2020). Comparison of the genefinderTM COVID-19 plus realAmp kit on the sample-to-result platform ELITe ingenius to the national reference method: an added value of N gene target detection?. *Journal of Clinical Virology*.

[22] Fukasawa L. O., Sacchi C. T., Gonçalves M. G., Lemos A. P. S., Almeida S. C. G., Caterino-de-Araujo A. (2021). Comparative performances of seven quantitative reverse-transcription polymerase chain reaction assays (RT-qPCR) for detecting SARS-CoV-2 infection in samples from individuals suspected of COVID-19 in são paulo, brazil. *Journal of Clinical Virology*.

